# A New Combined Air Quality and Heat Index in Relation to Mortality in Monterrey, Mexico

**DOI:** 10.3390/ijerph19063299

**Published:** 2022-03-11

**Authors:** Shayna K. Fever, Jonathan D. W. Kahl, Amy E. Kalkbrenner, Rosa M. Cerón Bretón, Julia G. Cerón Bretón

**Affiliations:** 1 Atmospheric Science Program, University of Wisconsin-Milwaukee, Milwaukee, WI 53211, USA; shayna.fever@gmail.com; 2 Zilber School of Public Health, University of Wisconsin-Milwaukee, Milwaukee, WI 53211, USA; kalkbren@uwm.edu; 3 Department of Chemistry, Autonomous University of Carmen, Campeche 24180, Mexico; rosabreton1970@gmail.com (R.M.C.B.); jceronbreton@gmail.com (J.G.C.B.)

**Keywords:** air quality index, heat index, combined index, air quality–mortality relationships, heat-mortality relationships

## Abstract

The negative synergistic effects of air pollution and sensible heat on public health have been noted in numerous studies. While separate, simplified, and public-facing indices have been developed to communicate the risks of unhealthful levels of air pollution and extreme heat, a combined index containing elements of both has rarely been investigated. Utilizing air quality, meteorology, and mortality data in Monterrey, Mexico, we investigated whether the association between the air quality index (AQI) and mortality was improved by considering elements of the heat index (HI). We created combined indices featuring additive, multiplicative, and either/or formulations and evaluated their relationship to mortality. Results showed increased associations with mortality for models employing indices that combined the AQI and the HI in an additive or multiplicative manner, with increases in the interquartile relative risk of 3–5% over that resulting from models employing the AQI alone.

## 1. Introduction

Air pollution is a global environmental issue that poses a major threat to public health. Worldwide, approximately seven million people die each year from exposure to air pollution [1]. In order to communicate air quality to the public and to draw attention to its effects on human health, in 1976, the United States Environmental Protection Agency (EPA) developed the air quality index (AQI) [2]. Similar indices, such as Canada’s Air Quality Health Index [3], are in use in other countries.

**Figure 1:**
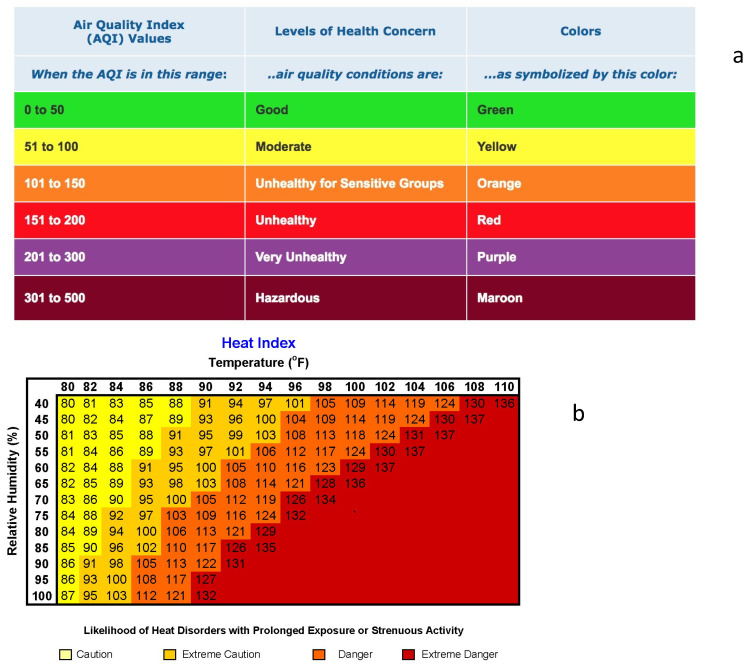
(**a**) Air quality index (AQI) chart (airnow.gov/aqi/aqi-basics, accessed on 1 March 2022), (**b**) heat index (HI) chart (www.weather.gov/phi/heatcond, accessed on 1 March 2022).

The AQI is based on five criteria air pollutants that are regulated under the United States Clean Air Act: sulfur dioxide (SO_2_), nitrogen dioxide (NO_2_), carbon monoxide (CO), ozone (O_3_), and particulate matter (PM_2.5_ and PM_10_). The AQI is communicated as six color-coded levels of increasing pollutant levels and health concern, from “good” in green to “hazardous” in maroon (Figure 1a). The negative health impacts of air quality are of particular concern for sensitive groups including those with heart and lung diseases, older adults, and children. The color-coded chart is intended to simplify the interpretation of the index values in order to facilitate public understanding of whether air pollution is reaching unhealthy levels.

Exposure to extreme heat also poses a threat to public health, with risks including heat-related illness, death, and exacerbation of preexisting chronic conditions [4,5,6,7,8]. The human-perceived sense of heat depends on both relative humidity and temperature, as quantified by the heat index (HI) [9,10]. Similarly to the AQI, a categorical heat index chart is utilized to communicate the potential danger of extreme heat to the public, with color-coded categories corresponding to different levels of danger (Figure 1b).

Air pollutant and heat exposures together may act synergistically to increase the harm from either individually, despite the fact that these risks are communicated to the public separately via the AQI and HI [11,12]. In urban areas in particular, heat exposure and high pollution levels are the two most dangerous factors that simultaneously affect local populations [13]. The interaction between these environmental risk factors is predicted by some shared biologic pathways, such as impacts on the respiratory and cardiac systems. There is human data support of such interactions from various parts of the world: Cerón-Bretón et al. [14] reported higher mortality rates associated with elevated pollutant concentrations, with even stronger associations in simulated climate warming scenarios. Filleul et al. [15] reported that the joint effects of ozone pollution and high temperatures resulted in a significant increase in the risk of death in nine urban areas across France. In Beijing, China, Zhang et al. [16] found particulate matter to be significantly associated with daily mortality from respiratory and cardiovascular causes. These associations were amplified during periods of high temperatures, suggesting that controlling the particulate emissions during warm months could substantially benefit population health. Yilmaz et al. [17] noted significant correlations between air pollution, thermal comfort, and urban morphology in Erzurum, Turkey. The studies cited above, conducted in diverse parts of the world with different climate conditions, exemplify the potential negative synergistic effect of air pollution and sensible heat (i.e., heat that can be felt) on public health. Simultaneously considering air pollution and heat is further underscored by the fact that high air pollutants and extreme heat often co-occur, in part due to the role of photochemistry in ozone formation.

While separate, simplified indices have been developed to communicate the risks of unhealthful levels of air pollution and extreme heat to the public, very few studies have addressed the combined effects of both environmental risk factors [17,18]. Yilmaz et al. [17] focused on the interrelationships between urban microclimates, urban morphology, and air pollution in a cold, high altitude city in Turkey. Steeneveld et al. [19] developed a metric for northwestern Europe that combines the effects of high temperatures and high pollution concentrations at neighborhood scales. This combined metric was designed for use as an urban climate management tool and can be used to assess the impacts of air quality and the urban heat island on human health. Susca and Pompani [20] reviewed life cycle assessment (LCA) studies in urban areas. They noted that interactions between local climates and built environments, particularly the HI, were frequently reported and suggested that additional indicators, including urban air pollution, be integrated into the LCA methodology. It has also been noted, however, that different research communities, e.g., urban planning, land use, public policy, building science, and thermal comfort, have different perspectives on coping with heat vulnerabilities and their efforts are often not aligned [21,22].

The need for a combined air quality and heat index has been clearly articulated by Pascal et al. [23] and is strengthened by the growing prevalence of high heat exposure associated with the increasing pressures of anthropogenic climate change [24]. To evaluate the usefulness of a combined index in predicting health impacts directly on human data, here we investigate whether the association between the AQI and mortality can be improved by incorporating elements of the HI. While decades of chronic air pollutant and heat exposures can certainly influence cardiovascular and respiratory health, here we focus on their short-latency impacts on mortality; these are important health relationships which are highly relevant to public health index reporting systems. We will show that a single index which includes both the AQI and HI can be an effective metric for communicating the combined risks of air pollution and extreme heat to the public.

## 2. Materials and Methods

### 2.1. Data

We evaluated associations between several combined air quality and heat indices and mortality, using real data from Monterrey, Mexico.

We used meteorological, air quality, and mortality data collected from 2012–2015 in the metropolitan area of Monterrey, Mexico (25.67° N, 100.30° W). The dataset is described briefly here; full details are provided by Cerón-Bretón et al. [14]. Monterrey, the capital of the state of Nuevo León, and a sprawling business and industrial center, has a total population of over 5.3 million residents and a land area of 5407 km^2^ [25]. The climate is warm-semiarid (Figure S1 in Supplemental Information).

We included data on people residing in Monterrey, Mexico, who died during the warm months of 2012–2015. We only included deaths in warm months, defined as 1 July–31 October, to reduce the effect of annual cyclical trends in pollution concentrations, temperature, relative humidity, and mortality. Additionally, the synergistic interactions of extreme heat and AQI on mortality would be expected to be most evident in the warm season. We only included deaths categorized as respiratory- or cardiovascular-related as the causes of such deaths are known to be related to adverse effects of air pollution and extreme heat [26,27,28,29,30]. Accidental deaths were excluded. Mortality data was obtained from the Mexican National Health Information System (www.dgis.salud.gob.mx, accessed on 1 March 2022). Additional details are provided in Cerón-Bretón et al. [14].

We used hourly measurements of criteria air pollutant concentrations, temperature, and relative humidity collected at air quality monitoring sites in each of the eight Monterrey municipalities for which data were available (Figure S1). The air quality and meteorological data were obtained from Sistema Integral de Monitoreo Ambiental, the air quality monitoring network of Nuevo León (aire.nl.gob.mx, accessed on 1 March 2022). Quality control measures applied to the mortality, air pollution, and meteorological data fields are described by Cerón-Bretón et al. [14].

### 2.2. Methods

Using Poisson regression models to incorporate different combinations of air quality and heat indices, we identified associations between these environmental risk factors and mortality within vulnerable population sets and quantified these associations using interquartile relative risk. The associations resulting from combined indices were compared with those of baseline models to evaluate whether combinations of air quality and heat indices increased the strength of association with mortality.

Our approach involved an ecological design with human data grouped by time, for each day during our study period, and linked to air pollutant and meteorological information on the same day. We allowed for up to 7 days for heat and air pollution to impact mortality by employing lags in our models. These lag times are consistent with previous understandings of the acute impacts of air pollution, heat, and their interaction on mortality, for example [31,32]. Mortality data were aggregated over the entire Monterrey study area, rather than being linked to air pollution at a finer spatial level because we assumed that temporal changes in heat and air pollutants were more important in driving mortality patterns. Furthermore, risk communication of air quality and heat indices is often homogeneous across such a metropolitan area [33].

#### 2.2.1. Calculating AQI Values

Daily, pollutant-specific AQI values were calculated in each municipality for each criteria air pollutant (hourly PM_2.5_ measurements were available from only two of the eight municipalities (Guadalupe and Santa Catarina) in 2012–2014 and only from Santa Catarina in 2015). Following the U.S. EPA procedure [2], averaging times were based on the U.S. National Ambient Air Quality Standards: 1 h averages for NO_2_ and SO_2_, 8 h averages for CO and O_3_, and 24 h averages for PM_2.5_ and PM_10_. To represent the general (i.e., overall) AQI for the entire metropolitan area we selected the highest pollutant-specific AQI from among the eight municipalities, as individual pollutant time series at the eight monitoring sites tended to be similar among all the municipalities (Figures S2–S7 in Supplemental Information).

Since levels of PM_2.5_ and O_3_ were frequently high and were often the pollutant-specific AQI values selected to represent the general AQI, we also examined pollutant-specific AQI values for PM_2.5_ and O_3_ in our models with mortality.

#### 2.2.2. Calculating Heat Index Values

Daily HI values were determined for each municipality using the algorithm developed by the National Weather Service [10]. Each day’s highest hourly temperature measurement and that hour’s measured relative humidity were used in the calculations. In parallel to the AQI, we selected the highest HI value among the eight municipalities as the daily HI value for the Monterrey metropolitan area, as HI time series at the eight monitoring sites were largely similar (Figure S8 in Supplemental Information).

#### 2.2.3. Developing a Transformed Heat Index

Using a procedure similar to that by which individual pollutant concentrations are transformed into the AQI [2], we converted the HI into a form with a scale consistent with that of the AQI. Two versions of the transformed HI were created, *I_HI_A_* and *I_HI_B_*, differing in the breakpoints used to delineate ranges of health concern (see Transformed Heat Index, Supplemental Information, for full details). These transformed indices were used in the formulations for the combined air quality and heat indices (Section 2.2.4).

#### 2.2.4. Developing Combined Air Quality and Heat Indices

We constructed and tested three types of combined air quality and heat indices. Each type combined these environmental risk factors in a different way (Table 1).

The first type, termed “either/or” indices, followed the standard AQI methodology [2] where the largest of several individual pollutant AQIs was designated as the general AQI. The NEW model took the higher of the transformed heat index *I_HI_A_* or *I_HI_B_* value and each of the three AQI values each day (general AQI, PM_2.5_ AQI, and O_3_ AQI), thereby allowing one aspect (heat or air pollution) to override the other. NEW-A index values were the higher of daily AQI and *I_HI_A_* values; NEW-B index values were the higher of daily AQI and *I_HI_B_* values.

The second type of combined index was multiplicative, consisting of the product of an AQI (general AQI, PM_2.5_ AQI, and O_3_ AQI) and a heat index. Three multiplicative indices were constructed. MULT-HI was the product of AQI and HI; MULT-HI_A was the product of AQI and the transformed heat index *I_HI_A_*, and MULT-HI_B was the product of AQI and the transformed heat index *I_HI_B_*.

The final type of combined index examined was additive, consisting of the sum of an AQI (general AQI, PM_2.5_ AQI, and O_3_ AQI) and a heat index. The three additive indices examined were SUM-HI, the sum of AQI and HI; SUM-HI_A, the sum of AQI and the transformed heat index *I_HI_A_*; and SUM-HI_B, the product of AQI and the transformed heat index *I_HI_B_*.

#### 2.2.5. Vulnerable Population Sets

We considered deaths for all persons and deaths restricted to persons >65 years separately because persons >65 are more vulnerable to the adverse effects of air pollution and extreme heat. The mean (standard deviation) number of daily deaths due to respiratory or cardiovascular reasons was 12.5 (3.9) for persons >65 years, and 15.6 (4.4) for all persons.

#### 2.2.6. Poisson Regression Models

To analyze the relationship of daily warm-month mortality with respect to explanatory variables, we constructed ten Poisson regression models according to common methodologies used in previous studies [14,34,35]. In the Poisson model, daily mortality *M* is predicted according to

(1) $$ln\left(M\right)=\beta_{0}+\sum_{i=1}^{n}\beta_{i}x_{i}$$

where *x* is the explanatory variable(s), *β* is the regression coefficient(s) with intercept *β*_0_, and *n* is the number of explanatory variables.

Model AQI-only (Table 1) utilizes each version of the daily AQI (general, PM_2.5_, and O_3_) as the sole predictor of daily mortality within each population set. This serves as the baseline model against which improvements in the associations between response and explanatory variables (i.e., a combined index) are measured. Model AQI+HI includes the (untransformed) heat index as a second explanatory variable. The AQI+HI model, the only model tested with two explanatory variables, was utilized to assess the improvement in the association when the HI is considered as an additional explanatory variable, as opposed to a component of a combined index.

In all models, explanatory variables were normalized by their respective standard deviations. Seasonality was controlled by restricting analysis to warm months (July to October). Controls for long term trends were not applied, as the pollutant and heat index time series revealed no apparent trends (Figures S2–S8 in Supplemental Information). Response variables for all models were lagged by 0–7 days, as many previous studies have reported that the effects of air pollution and extreme heat on daily mortality is not necessarily immediate [36] and can occur at lags of 20 days or longer [34]. To ensure the robustness of our statistical analyses, we generated 1000 synthetic datasets for each regression model run using the bootstrapping method [37]. Calculations were done using the MATLAB software function *fitglm*.

Associations between explanatory and response variables were quantified using interquartile relative risk (*RR*) [38], defined as

(2) $$RR=\frac{M_{75}}{M_{25}}$$

where *M*_75_ and *M*_25_ are predicted mortality according to Equation (1), utilizing the 75th and 25th percentiles, respectively, of the explanatory variable(s).

The *RR* was determined as the average of the individual *RR*s corresponding to each of the 1000 bootstrapped regressions performed for each model run. The full range of 1000 *RR*s was used to determine statistical significance and 95% confidence intervals. A *RR* value was considered to be statistically significant if at least 95% of the synthetic *RR* values were >1.0. Confidence intervals were reported as [*a*, *b*] where *a* and *b* are the 2.5th and 97.5th percentiles, respectively, of the *RR* distributions.

#### 2.2.7. Change in Relative Risk: Comparison with Baseline Model

Since our goal was to evaluate whether new types of indices combining air quality and heat information provided stronger associations with mortality, we directly compared *RR*s from the new indices and the baseline model using only the AQI. We selected models from lag times of 3 and 4 days because these lags exhibited the largest *RR*s (shown later in Section 3). We calculated an improvement by subtracting the central *RR* estimates from models being compared: Δ Relative Risk (Δ*RR*),

(3) $$\Delta RR=RR_{lag}^{model}-RR_{lag}^{baseline}$$

where $RR_{lag}^{model}$ is the interquartile relative risk associated with a particular model, AQI type, population set, and lag and $RR_{lag}^{baseline}$ is the relative risk corresponding to the baseline AQI-only model for the same AQI type, population set, and lag.

## 3. Results

### 3.1. Air Quality Index (AQI) and Heat Index (HI) in Monterrey

**Figure 2:**
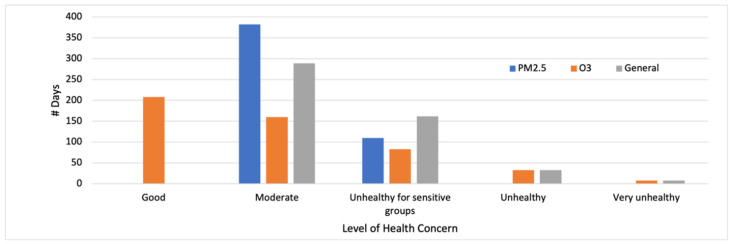
Number of days during warm months, Monterrey, Mexico, 2012–2015, for which different levels of health concern were indicated by the general, PM_2.5_, and O_3_ AQI.

Air quality standards are frequently exceeded in Monterrey, principally due to PM_2.5_ and O_3_ [39,40,41]. During the 492 days in the warm months of 2012–2015, the daily general AQI never indicated “good” air quality (Figure 2). Elevated levels of *both* PM_2.5_ and O_3_ frequently occurred during this period. There were 284 days during which the PM_2.5_ and O_3_ AQIs both indicated “moderate” or more severe health concerns. On 37 days the PM_2.5_ and O_3_ AQIs both indicated levels of health concern of “unhealthy for sensitive groups” or worse.

The PM_2.5_ AQI and O_3_ AQI were thus designated as the general AQI on 250 (51%) and 167 (34%), respectively, of the 492 days analyzed (Table 2). The O_3_ AQI was designated as the general AQI on the 41 days in which the AQI health category was “unhealthy” or “very unhealthy”. Both of these classifications carry cautionary guidance, including the avoidance of prolonged physical activity for people with heart or lung disease, older adults, and other sensitive groups [2].

The HI level of concern was categorized as “caution” on 98 (20%) days, and as “extreme caution”, “danger”, or “extreme danger” on 377 (77%) days. The high frequency of elevated HI values suggests that heat-mortality associations [3] are likely to be apparent in Monterrey.

### 3.2. Interquartile Relative Risk: Baseline AQI-Only and AQI+HI Model

**Figure 3:**
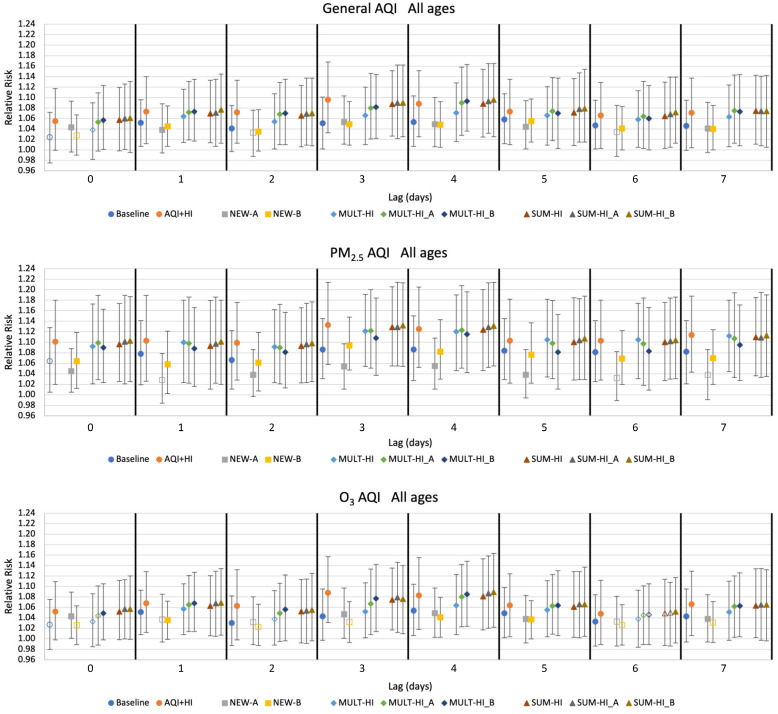
The interquartile relative risks and 95% confidence intervals for all models (deaths of persons of all ages). Model characteristics are described in Table 1. Solid symbols indicate significance at *p* = 0.05.

**Figure 4:**
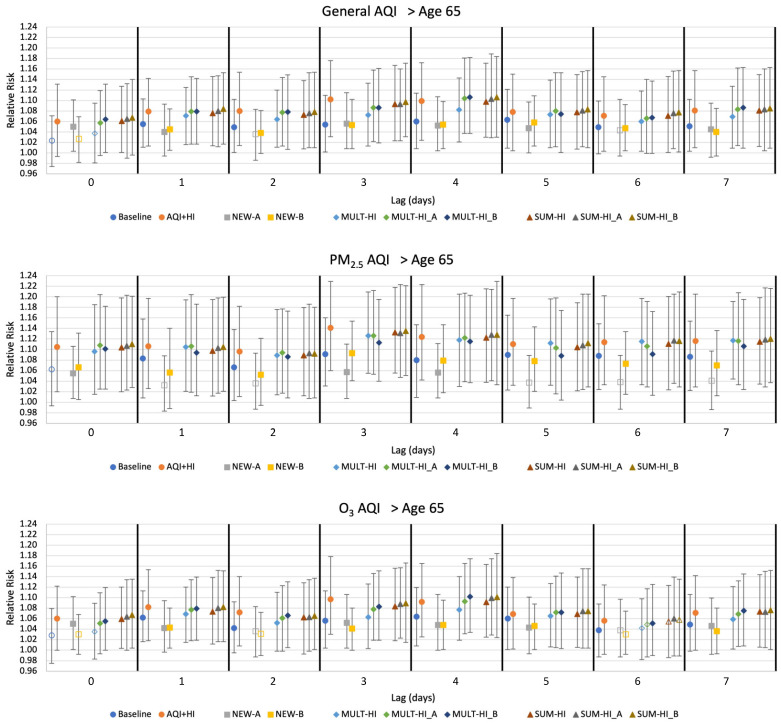
The interquartile relative risks and 95% confidence intervals for all models (deaths of persons >65 years). Model characteristics are described in Table 1. Solid symbols indicate significance at *p* = 0.05.

Interquartile relative risk (*RR*) results for all models are shown in Figure 3 for deaths in all persons, and Figure 4 for deaths in persons >65 years. Results are presented for lags ranging from zero to seven days. Because associations were the strongest for lags of three and four days, we limit tabular *RR* results to these lags (Tables S3 and S4, Supplemental Information).

Baseline AQI-only model results corroborate that the known AQI-mortality relationships [27] are clearly evident in Monterrey in these data. The *RR* associated with all three AQI types are significantly associated with the mortality for all lags ≥1 day with values ranging from 1.030 to 1.091. *RR*s were similar when including deaths of all ages and when restricted to deaths in persons >65 years. The presence of these AQI-mortality relationships in our Monterrey dataset allowed us to test whether such associations could be made stronger by including elements of the HI in the regressions.

When the HI was included as a second explanatory variable together with the AQI (in the AQI+HI model), the association between mortality and these environmental factors was stronger than the association between mortality and AQI alone. This result applies to all lags for deaths of persons of all ages and for those >65 years and all AQI types, with RRs ranging from 1.048 to 1.141. *RR* values for deaths in persons >65 years were slightly larger, consistent with the known vulnerabilities of individuals above the age of 65 with cardiovascular- or respiratory-related conditions [27].

### 3.3. Interquartile Relative Risk: Models with Either/or Indices

The NEW-A and NEW-B models each feature either/or type indices as their explanatory variable (Table 1), defined as the higher of the transformed heat index *I_HI_A_* or *I_HI_B_* value and each form of the three AQI values each day (general AQI, PM_2.5_ AQI, and O_3_ AQI).

The relative risks associated with the NEW-A and NEW-B models were rarely larger than that of the AQI only model (Figure 3 and Figure 4; Tables S3 and S4). *RR* values nevertheless ranged from 1.035 to 1.094, indicating positive associations between these indices and mortality. A combined index using an either/or framework is thus positively associated with mortality in Monterrey but does not strengthen the association between mortality and AQI alone.

### 3.4. Interquartile Relative Risk: Models with Multiplicative Indices

The MULT-HI, MULT-HI_A, and MULT-HI_B models each utilize a combined index defined as the product of the AQI (general AQI, PM_2.5_ AQI, and O_3_ AQI) and the heat index (the untransformed HI, or the transformed *I_HI_A_* or *I_HI_B_*) as single explanatory variables (Table 1).

Interquartile relative risks associated with the multiplicative index models were nearly always larger than those associated with the baseline AQI-only model. RRs for these models were largest when using the PM_2.5_ AQI with values ranging from 1.081–1.126 (Figure 3 and Figure 4; Tables S3 and S4). Little difference in *RR* values was observed among multiplicative indices constructed with different HI components.

### 3.5. Interquartile Relative Risk: Models with Additive Indices

The SUM-HI, SUM-HI_A, and SUM-HI_B models each utilize a combined index consisting of the sum of the AQI (general AQI, PM_2.5_ AQI, and O_3_ AQI) and the heat index (the untransformed HI, or the transformed *I_HI_A_* or *I_HI_B_*) as a single explanatory variable (Table 1).

For all lags and AQI types, as well as for both population sets, the additive index models displayed *RR* values that were larger than those associated with the baseline AQI-only model (Figure 3 and Figure 4; Tables S3 and S4). As with the multiplicative index models, *RR* values were highest when the combined indices utilized the PM_2.5_ AQI with values ranging from 1.089–1.135. Smaller values, ranging from 1.049–1.102, were observed when using the O_3_ AQI. Little difference in *RR* values was observed among the three additive indices created with different HI components.

### 3.6. Change in Relative Risk: $\Delta RR=RR_{lag}^{model}-RR_{lag}^{baseline}$

**Figure 5:**
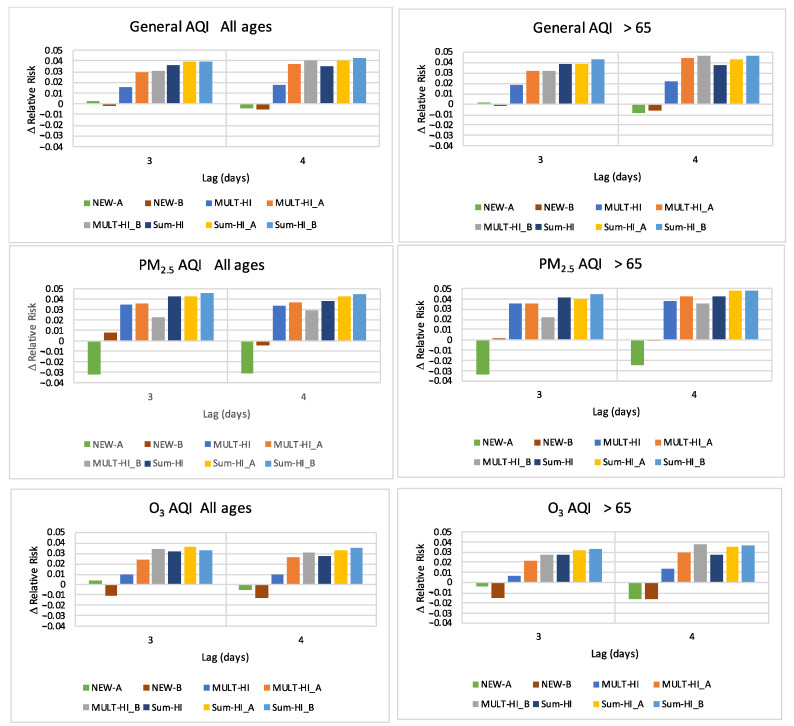
The increase in the interquartile relative risk over the baseline (AQI-only) model, for lags of three and four days. Model characteristics are described in Table 1.

Direct numerical comparisons of the magnitude of *RR*s are shown in the Δ*RR* results (Figure 5). When multiplicative or additive combined air quality and heat indices were considered, the interquartile relative risk was around 3–5% larger than that resulting from the AQI alone. Combined indices based on an either/or algorithm (the NEW-A and NEW-B models) did not yield increases in the association between mortality and environmental risk factors of air quality and extreme heat beyond those based on AQI alone.

The new additive index SUM-HI_B, that combined AQI with the transformed HI *I_HI___B_*, is shown in light blue in Figure 5. This index provided the highest *RR* values compared to using the AQI alone, across lag times of 3 and 4 days and across AQI using PM_2.5_ alone, O_3_ alone, or the highest pollutant value (general AQI).

The observed increase in *RR* provided by models utilizing combined air quality and heat indices is consistent with the extensive literature describing multiple biological pathways leading to deaths from respiratory and cardiovascular causes [32]. Examples include pulmonary inflammation and oxidative stress leading to hemostasis response [42] and pollution-mediated thrombosis through oxidative stress in concert with circulating microvesicles [43], among others.

## 4. Conclusions

Our results are generally consistent with prior publications which describe negative, synergistic short-term health impacts of air pollution and heat exposures. A unique aspect of this work is the development and testing of several types of combined air quality and heat indices, with their application to mortality in a major metropolitan zone. The results offer evidence that a combined index can communicate the interacting effects of air pollution and heat more effectively than separate indices.

**Figure 6:**
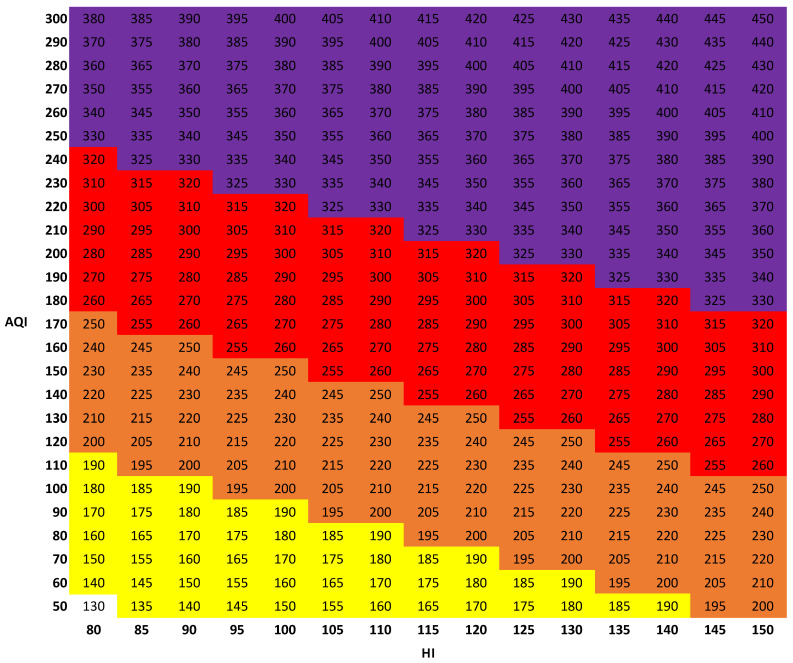
Chart depicting the combined air quality and heat index SUM-HI (index details are described in Table 1).

Indices that combine air quality and heat information in additive and multiplicative manners provided notable improvements in predicting mortality. A chart depicting one such additive index is shown in Figure 6. The color of each region of health concern corresponds to the ranges described in Figure 1. The chart is similar to the “urban climate index” for northwestern Europe [19]. Such a graphic, along with public communication of AQI and HI values, would allow citizens to readily determine the level of health concern corresponding to a combined air quality and heat index.

To fully evaluate the robustness of our new combined indices across situations, we examined several scenarios. We considered air quality for the most common pollutants in the area studied, PM_2.5_ and ozone, and also considered a general AQI selected from among the highest value of six criteria pollutants. We considered several lag times allowing for the delayed biologic impact of pollutant exposures. We evaluated different means of categorizing heat severity. We considered deaths among all persons and also considered a more vulnerable group—deaths in persons >65 years of age. Our primary findings—that the additive and multiplicative indices provided improvements in prediction of mortality—were consistent across these many factors, providing strength to this body of evidence.

Yet the performance of these new combined indices may not be generalizable to regions with very different air pollutant and weather patterns. Specifically, Monterrey, Mexico, exhibited numerous days of poor air quality, especially for PM_2.5_. Areas with lower pollutant levels, seasonal trends, and even different population-level susceptibilities to the effects of heat or air quality may experience different patterns of risk in relation to these combined air quality–heat indices.

Nevertheless, our results suggest that a single index which combines the AQI and HI in an additive or multiplicative manner could be an effective metric for communicating the combined risks of air pollution and extreme heat to the public. The utility of a combined index is consistent with epidemiologic evidence suggesting interacting effects of air pollution and heat, particularly in light of recent critical assessments of the single-pollutant AQI [44,45]. Further development and testing of a combined index using longer lags, cumulative lag days, or a longer-term data set, perhaps at neighborhood scales or for locations where humidity imparts a greater contribution to the heat index, could provide additional benefit. A future study conducted at municipal or neighborhood scales could provide useful information on the impact of microclimates and neighborhood-scale air quality on pollution/heat/mortality relationships. The importance of risk communication from the combined influences of air pollution and heat are likely to only grow given the pressures of climate change.

## Figures and Tables

**Table 1:** The characteristics of the Poisson regression models utilized.

Model Name	Explanatory Variables (Daily Values)
AQI-only (baseline)	AQI (general, PM_2.5_, or O_3_)
AQI+HI	AQI (general, PM_2.5_, or O_3_) *and* HI (This is the only model with two explanatory variables.)
**Either/or indices**	
NEW-A	AQI (general, PM_2.5_, or O_3_) *or* *I_HI_A_*
NEW-B	AQI (general, PM_2.5_, or O_3_) *or* *I_HI_B_*
**Multiplicative indices**	
MULT-HI	Product of AQI (general, PM_2.5_, or O_3_) and HI
MULT-HI_A	Product of AQI (general, PM_2.5_, or O_3_) and transformed heat index *I_HI_A_*
MULT-HI_B	Product of AQI (general, PM_2.5_, or O_3_) and transformed heat index *I_HI_B_*
**Additive indices**	
SUM-HI	Sum of AQI (general, PM_2.5_, or O_3_) and HI
SUM-HI_A	Sum of AQI (general, PM_2.5_, or O_3_) and transformed heat index *I_HI_A_*
SUM-HI_B	Sum of AQI (general, PM_2.5_, or O_3_) and transformed heat index *I_HI_B_*

**Table 2:** Number of days each individual air pollutant was the highest during warm months, Monterrey, Mexico, 2012–2015.

AQI ^a^ Health Category	Number of Days ^b^					
	SO_2_	NO_2_	CO	O_3_	PM_2.5_	PM_10_
Moderate	0	1	0	51	170	84
Unhealthy–Sensitive Groups	1	0	0	75	80	6
Unhealthy	0	0	0	33	0	0
Very Unhealthy	0	0	0	8	0	0

^a^ Abbreviations: AQI = air quality index, SO_2_ = sulfur dioxide, NO_2_ = nitrogen dioxide, CO = carbon monoxide, O_3_ = ozone, PM = particulate matter. ^b^ On 17 of the 492 days in the warm month dataset two pollutants had the same individual pollutant AQI as the general AQI. Either individual pollutant in these cases could be deemed the daily general AQI.

## Data Availability

Epidemiological data on mortality were obtained from the Mexico National Health Information System, www.dgis.salud.gob.mx, accessed on 1 March 2022. Air quality and meteorological data were obtained from the air quality monitoring network of Nuevo León, Mexico, http://aire.nl.gob.mx, accessed on 1 March 2022.

## Supplementary Materials

The following supporting information can be downloaded at: https://www.mdpi.com/article/10.3390/ijerph19063299/s1, Detailed description of the transformed heat index, Table S1: I*_HI-A_* breakpoints, Table S2: I*_HI-B_* breakpoints, Table S3: Interquartile relative risks and 95% confidence intervals for air quality and heat indices and respiratory and cardiovascular deaths at any age, Table S4: Interquartile relative risks and 95% confidence intervals for air quality and heat indices and respiratory and cardiovascular deaths at age > 65 years, Figure S1: Location of Monterrey, Mexico, and municipalities therein for which air quality, meteorological, and mortality data were available, Figure S2: Daily maximum time-averaged sulfur dioxide (SO_2_) concentrations by muncipality, Figure S3: Daily nitrogen dioxide (NO_2_) concentrations by muncipality, Figure S4: Daily carbon monoxide (CO) concentrations by muncipality, Figure S5: Daily ozone (O_3_) concentrations by muncipality, Figure S6: Daily particulate matter (PM_2.5_) concentrations by muncipality, Figure S7: Daily particulate matter (PM_10_) concentrations by muncipality, Figure S8: Daily Heat Index (HI) by muncipality.
