# Supplementary material for: A New Combined Air Quality and Heat Index in Relation to Mortality in Monterrey, Mexico
Source: Int J Environ Res Public Health. 2022 Mar 11;19(6):3299. doi: 10.3390/ijerph19063299 (PMC8948654; doi:10.3390/ijerph19063299)
Supplement: Supplementary file 1 [file ijerph-19-03299-s001.zip › ijerph-1585440-supplementary.pdf]

A New Combined Air Quality and Heat Index in Relation to Mortality in Monterrey, Mexico

Supplemental Information

Transformed Heat Index

Using a procedure similar to that by which individual pollutant concentrations are transformed into the AQI [2], we converted the HI into a form with a scale consistent with that of the AQI. The U.S. National Weather Service identifies four categories of concern related to HI value ranges: caution, extreme caution, danger, extreme danger (Figure 1b). We paired these HI ranges to the same index breakpoints that are used in four AQI ranges of concern: moderate, unhealthy for sensitive groups, unhealthy, and very unhealthy. The HI ranges and corresponding breakpoints for the first version of the transformed HI are presented in Table S1. Using these breakpoints, daily HI values were converted to transformed HI (I_{HI_A}) values using Eq. (S1),

$$I_{HI\_A} = \frac{I_{High} - I_{Low}}{BP_{High} - BP_{Low}} (V_{HI} - BP_{Low}) + I_{Low} \qquad (S1)$$

where V_{HI} is the daily HI value for the Monterrey, BP_{High} is the breakpoint that is greater than or equal to V_{HI}, BP_{Low} is the breakpoint that is less than or equal to V_{HI}, I_{High} is the HI value corresponding to BP_{High}, and I_{Low} is the HI value corresponding to BP_{Low}.

Constructing a transformed HI in this way resulted in a skewed distribution in which an inordinate number of days were classified as dangerous or extremely dangerous. In order to keep the frequency of the categorization of the transformed HI similar to that of the general, $PM_{2.5}$ and O_3 AQIs in our warm month dataset, we constructed a second version of a transformed HI, I_{HI_B},

using the same HI ranges as in I_{HI_A} but shifted down one category. I_{HI_B} values were also determined using Eq. (S1) with the revised breakpoints. These HI ranges and corresponding breakpoints are presented in Table S2.

Table S1. $I_{HI\text{-}A}$ breakpoints.

**HI values (°F)**	**Heat Index A (I_{HI_A}) Range**	**Concern**
**(BP_{Low}–BP_{High})**	**(I_{Low}–I_{High})**	
<80	0–50	No concern
80–90	51–100	Caution
91–103	101–150	Extreme caution
104–124	151–200	Danger
125–137	201–300	Extreme danger

Table S2. $I_{HI\text{-}B}$ breakpoints.

**HI values (°F)**	**Heat Index B (I_{HI_B}) Range**	**Concern**
**(BP_{Low}–BP_{High})**	**(I_{Low}–I_{High})**	
<80	0	No concern
80–90	0–50	Caution
91–103	51–100	Extreme caution
104–124	101–150	Danger
125–137	151–200	Extreme danger

Table S3. Interquartile relative risks and 95% confidence intervals for air quality and heat indices and respiratory and cardiovascular deaths at any age. All results are significant at p=.05 except where indicated.

Model[a]	General AQI[b]		$PM_{2.5}$ AQI		O_3 AQI	
	**Lag: 3 days**	**Lag: 4 days**	**Lag: 3 days**	**Lag: 4 days**	**Lag: 3 days**	**Lag: 4 days**
Baseline (AQI-only)	1.051 [1.002, 1.101]	1.053 [1.007, 1.103]	1.086 [1.031, 1.145]	1.086 [1.027, 1.150]	1.043 [0.997, 1.095]	1.054 [1.006, 1.104]
AQI+HI	1.096 [1.033, 1.169]	1.088 [1.025, 1.151]	1.133 [1.058, 1.214]	1.125 [1.052, 1.205]	1.088 [1.031, 1.157]	1.083 [1.018, 1.155]
Either/or models:						
NEW-A	1.053 [1.011, 1.113]	1.050 [1.006, 1.100]	1.054 [1.011, 1.097]	1.055 [1.011, 1.108]	1.047 [1.001, 1.097]	1.049 [1.003, 1.097]
NEW-B	1.049 [1.009, 1.092]	1.048 [1.005, 1.092]	1.093 [1.047, 1.148]	1.082 [1.030, 1.143]	1.032[c] [0.993, 1.071]	1.041 [1.003, 1.079]
Multiplicative models:						
MULT-HI	1.066 [1.010, 1.120]	1.071 [1.016, 1.128]	1.121 [1.054, 1.191]	1.120 [1.046, 1.190]	1.052 [1.002, 1.107]	1.064 [1.008, 1.123]
MULT-HI_A	1.080 [1.021, 1.146]	1.090 [1.028, 1.158]	1.122 [1.051, 1.200]	1.123 [1.051, 1.208]	1.067 [1.008, 1.133]	1.080 [1.023, 1.142]
MULT-HI_B	1.082 [1.022, 1.144]	1.093 [1.036, 1.163]	1.108 [1.037, 1.184]	1.115 [1.042, 1.196]	1.077 [1.014, 1.142]	1.085 [1.024, 1.148]
Additive models:						
SUM-HI	1.088 [1.027, 1.151]	1.088 [1.024, 1.154]	1.129 [1.055, 1.206]	1.124 [1.046, 1.200]	1.075 [1.017, 1.135]	1.081 [1.019, 1.153]
SUM-HI_A	1.090 [1.022, 1.162]	1.093 [1.032, 1.165]	1.129 [1.055, 1.214]	1.129 [1.052, 1.213]	1.079 [1.012, 1.146]	1.087 [1.020, 1.158]
SUM-HI_B	1.090 [1.025, 1.162]	1.096 [1.025, 1.165]	1.132 [1.054, 1.213]	1.131 [1.055, 1.214]	1.076 [1.010, 1.140]	1.089 [1.022, 1.163]

[a] Model details are provided in Table 1.

[b] Abbreviations: AQI = Air Quality Index, SO_2=sulfur dioxide, NO_2 = nitrogen dioxide, CO=carbon monoxide, O_3 = ozone, PM=particulate matter.

[c] Significant at p=.06.

Table S4. Interquartile relative risks and 95% confidence intervals for air quality and heat indices and respiratory and cardiovascular deaths at age > 65 years. All results are significant at p=.05.

**Model**[a]	**General AQI**[b]		**$PM_{2.5}$ AQI**		**O_3 AQI**	
	**Lag: 3 days**	**Lag: 4 days**	**Lag: 3 days**	**Lag: 4 days**	**Lag: 3 days**	**Lag: 4 days**
Baseline (AQI-only)	1.054 [1.002, 1.110]	1.060 [1.008, 1.114]	1.091 [1.031, 1.160]	1.080 [1.009, 1.147]	1.056 [1.004,1.113]	1.064 [1.009, 1.119]
AQI+HI	1.103 [1.031, 1.176]	1.099 [1.024, 1.172]	1.141 [1.060, 1.229]	1.124 [1.042, 1.223]	1.097 [1.029, 1.178]	1.092 [1.025, 1.165]
Either/or models:						
NEW-A	1.056 [1.008, 1.115]	1.052 [1.004, 1.107]	1.057 [1.007, 1.110]	1.056 [1.008, 1.111]	1.052 [1.004, 1.106]	1.048 [1.000, 1.106]
NEW-B	1.054 [1.008, 1.102]	1.054 [1.008, 1.098]	1.093 [1.041, 1.154]	1.079 [1.018, 1.147]	1.041 [1.000, 1.080]	1.048 [1.001, 1.095]
Multiplicative models:						
MULT-HI	1.072 [1.013, 1.133]	1.082 [1.021, 1.143]	1.126 [1.055, 1.209]	1.118 [1.030, 1.205]	1.063 [1.003, 1.126]	1.077 [1.019, 1.140]
MULT-HI_A	1.086 [1.022, 1.158]	1.104 [1.037, 1.181]	1.126 [1.053, 1.212]	1.122 [1.039, 1.207]	1.078 [1.019, 1.146]	1.093 [1.031, 1.165]
MULT-HI_B	1.086 [1.019, 1.161]	1.106 [1.037, 1.182]	1.113 [1.040, 1.195]	1.115 [1.037, 1.203]	1.083 [1.019, 1.151]	1.102 [1.034, 1.174]
Additive models:						
SUM-HI	1.093 1.023, 1.167]	1.098 [1.030, 1.171]	1.133 [1.055, 1.218]	1.123 [1.038, 1.215]	1.083 [1.018, 1.156]	1.092 [1.025, 1.163]
SUM-HI_A	1.093 [1.023, 1.160]	1.103 [1.029, 1.189]	1.131 [1.047, 1.223]	1.128 [1.041, 1.214]	1.088 [1.023, 1.157]	1.099 [1.029, 1.174]
SUM-HI_B	1.097 [1.031, 1.171]	1.106 [1.030, 1.184]	1.135 [1.051, 1.221]	1.128 [1.033, 1.229]	1.089 [1.015, 1.166]	1.101 [1.024, 1.184]

[a] Model details are provided in Table 1.

[b] Abbreviations: AQI = Air Quality Index, SO_2=sulfur dioxide, NO_2 = nitrogen dioxide, CO=carbon monoxide, O_3 = ozone, PM=particulate matter.

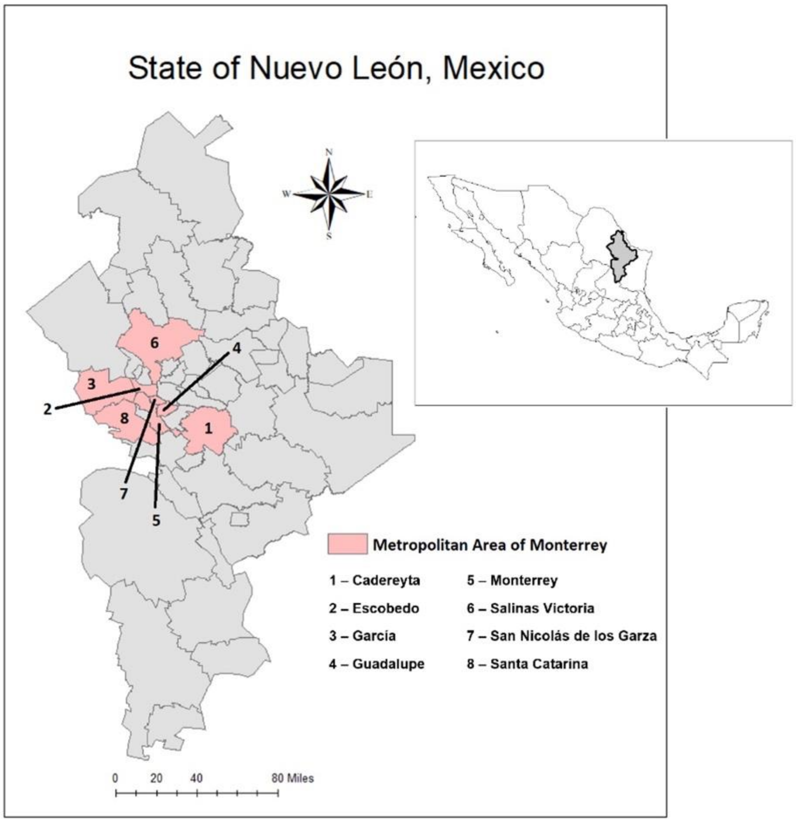

Figure S1. Location of Monterrey, Mexico, and municipalities therein for which air quality, meteorological, and mortality data were available.

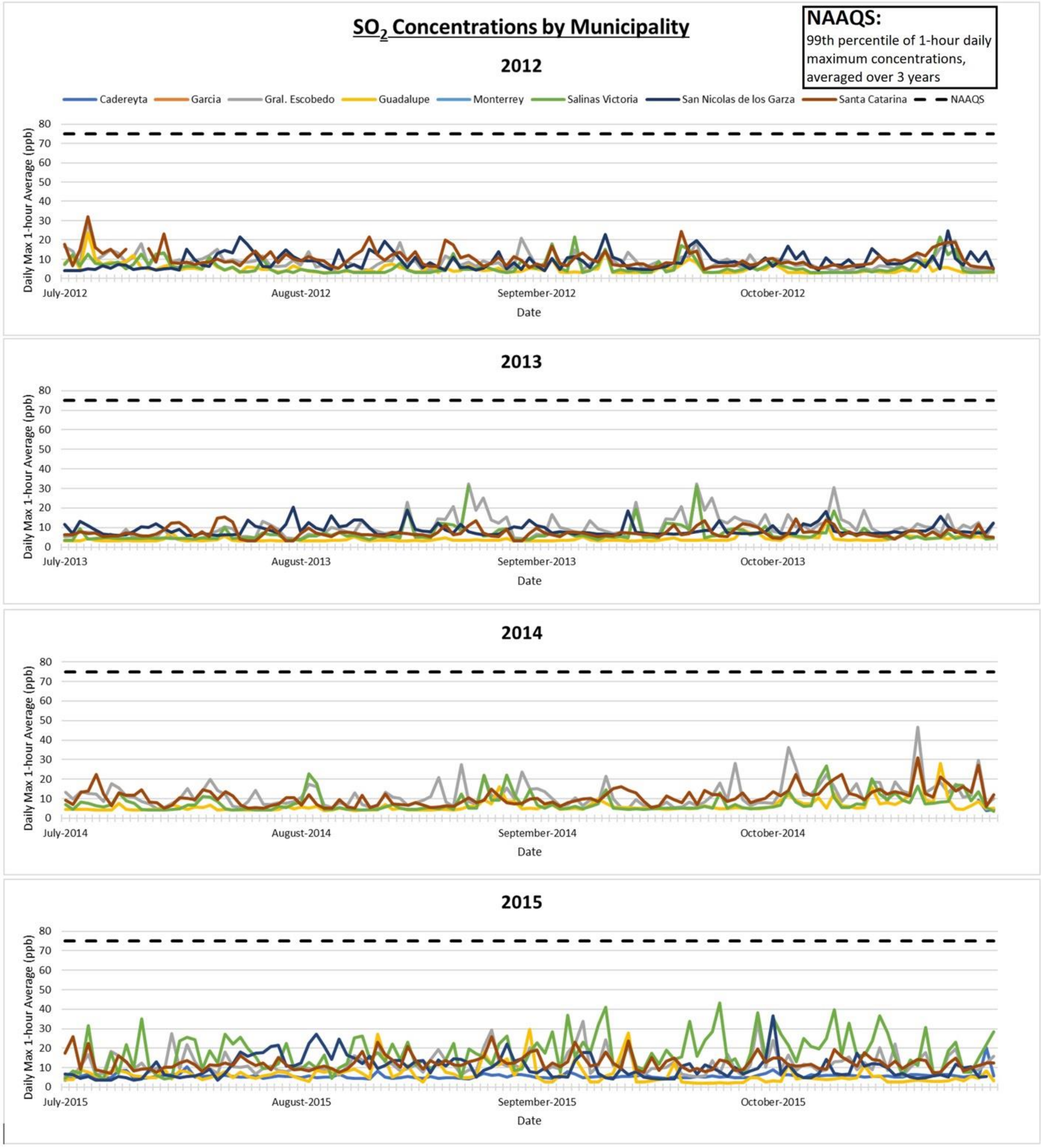

Figure S2. Daily maximum time-averaged sulfur dioxide (SO_2) concentrations by muncipality. Averaging times are based on the U.S. National Ambient Air Quality Standards [2].

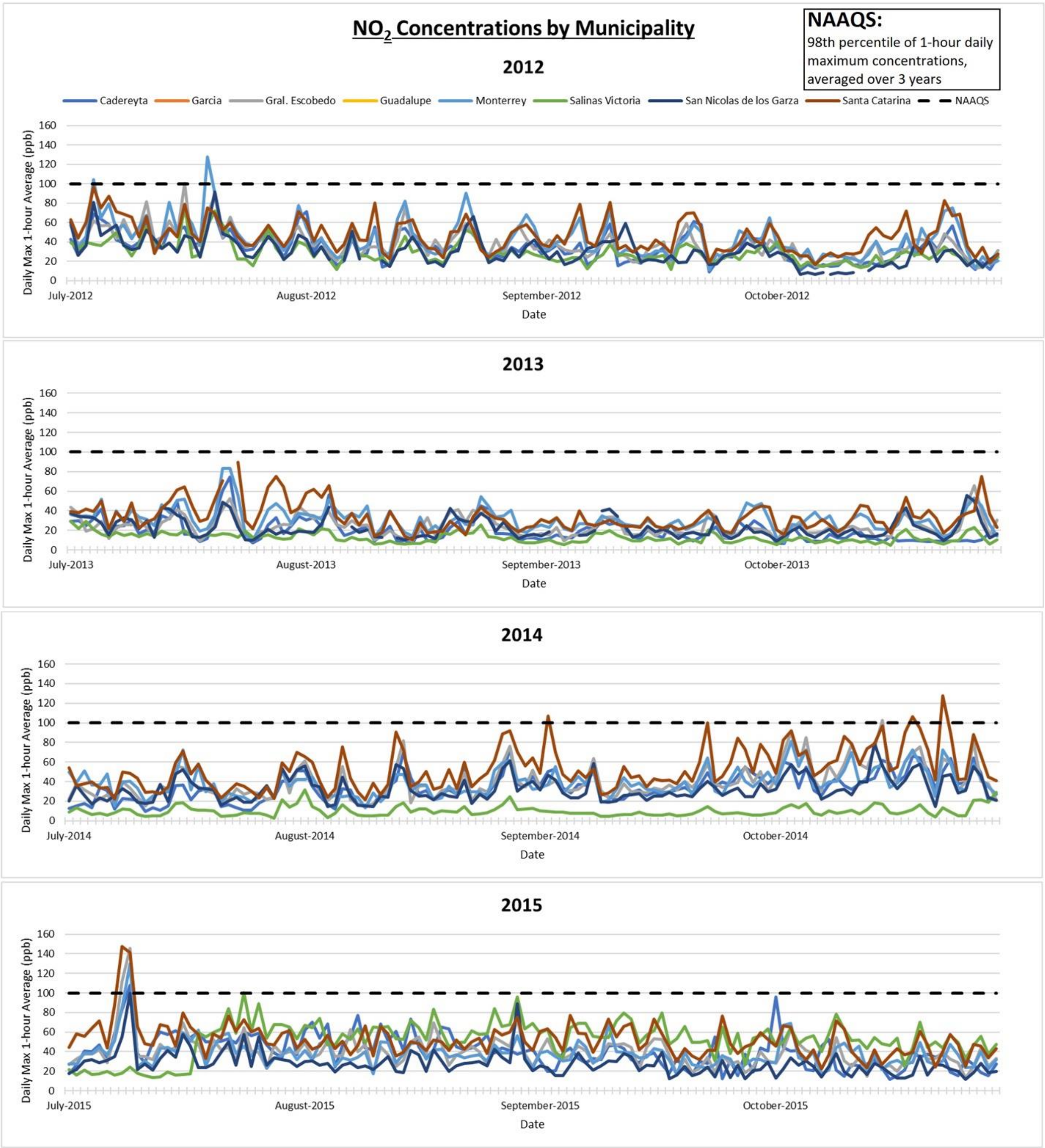

Figure S3. Daily nitrogen dioxide (NO_2) concentrations by muncipality. Averaging times are based on the U.S. National Ambient Air Quality Standards [2].

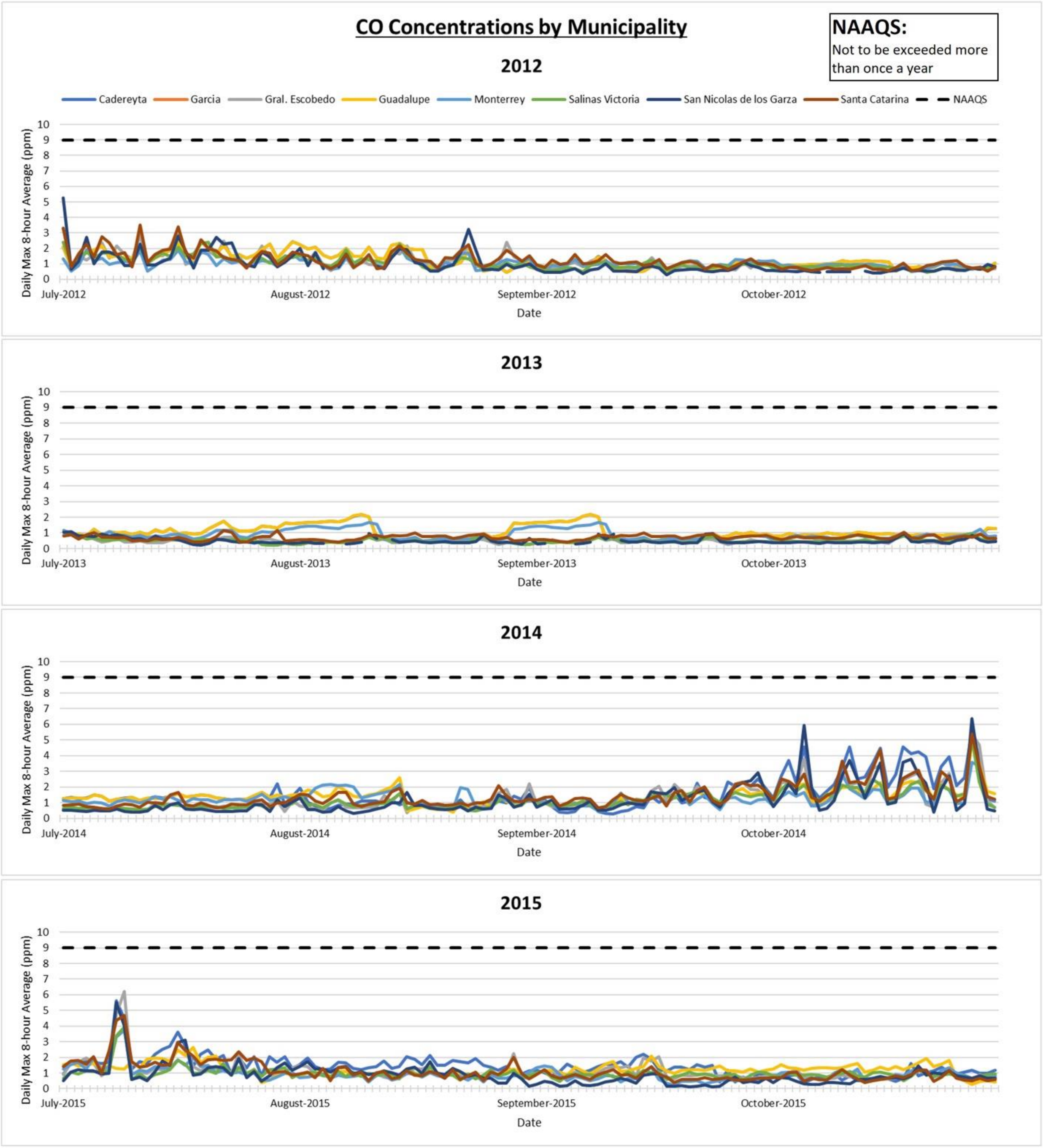

Figure S4. Daily carbon monoxide (CO) concentrations by muncipality. Averaging times are based on the U.S. National Ambient Air Quality Standards [2].

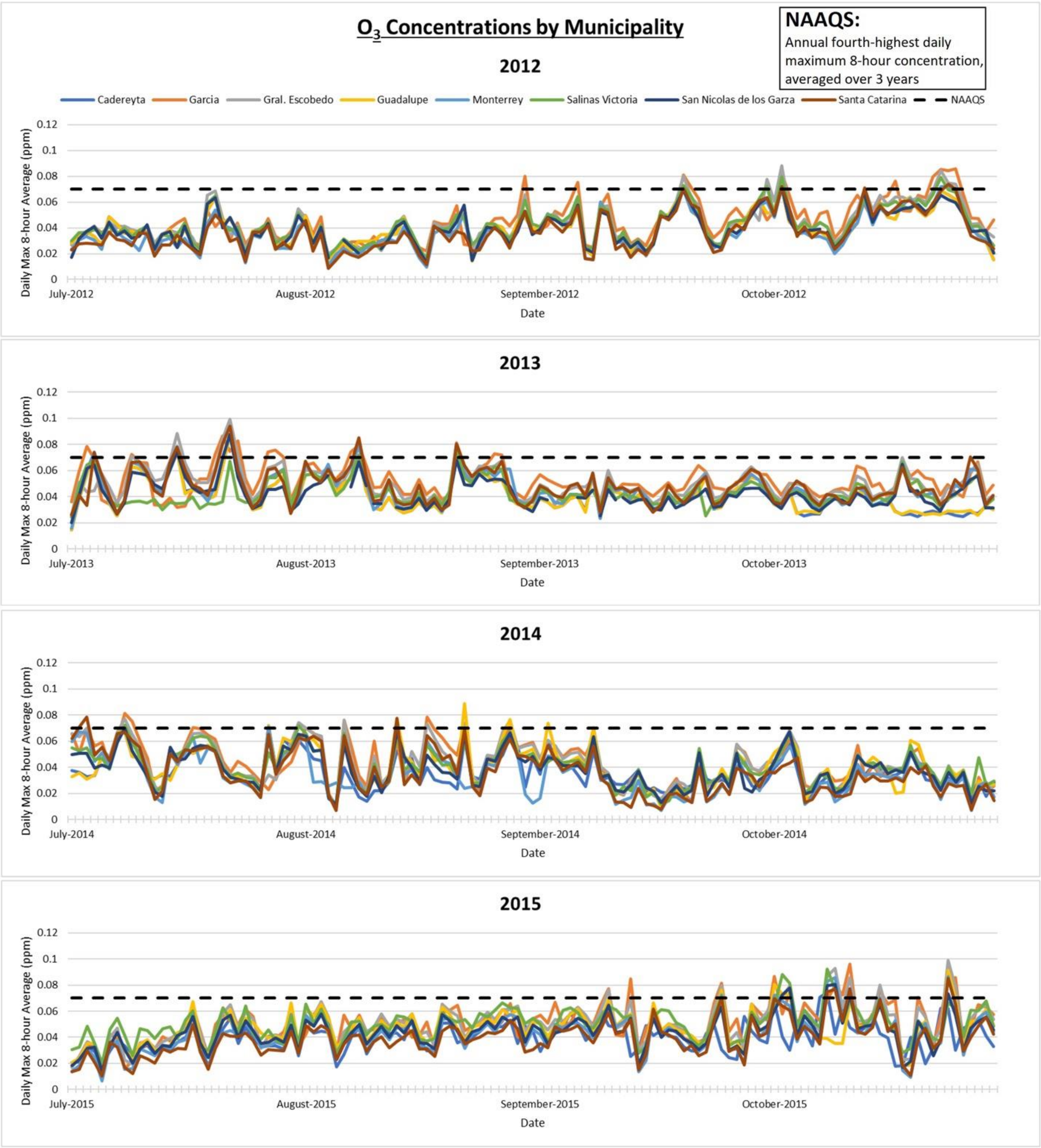

Figure S5. Daily ozone (O_3) concentrations by muncipality. Averaging times are based on the U.S. National Ambient Air Quality Standards [2].

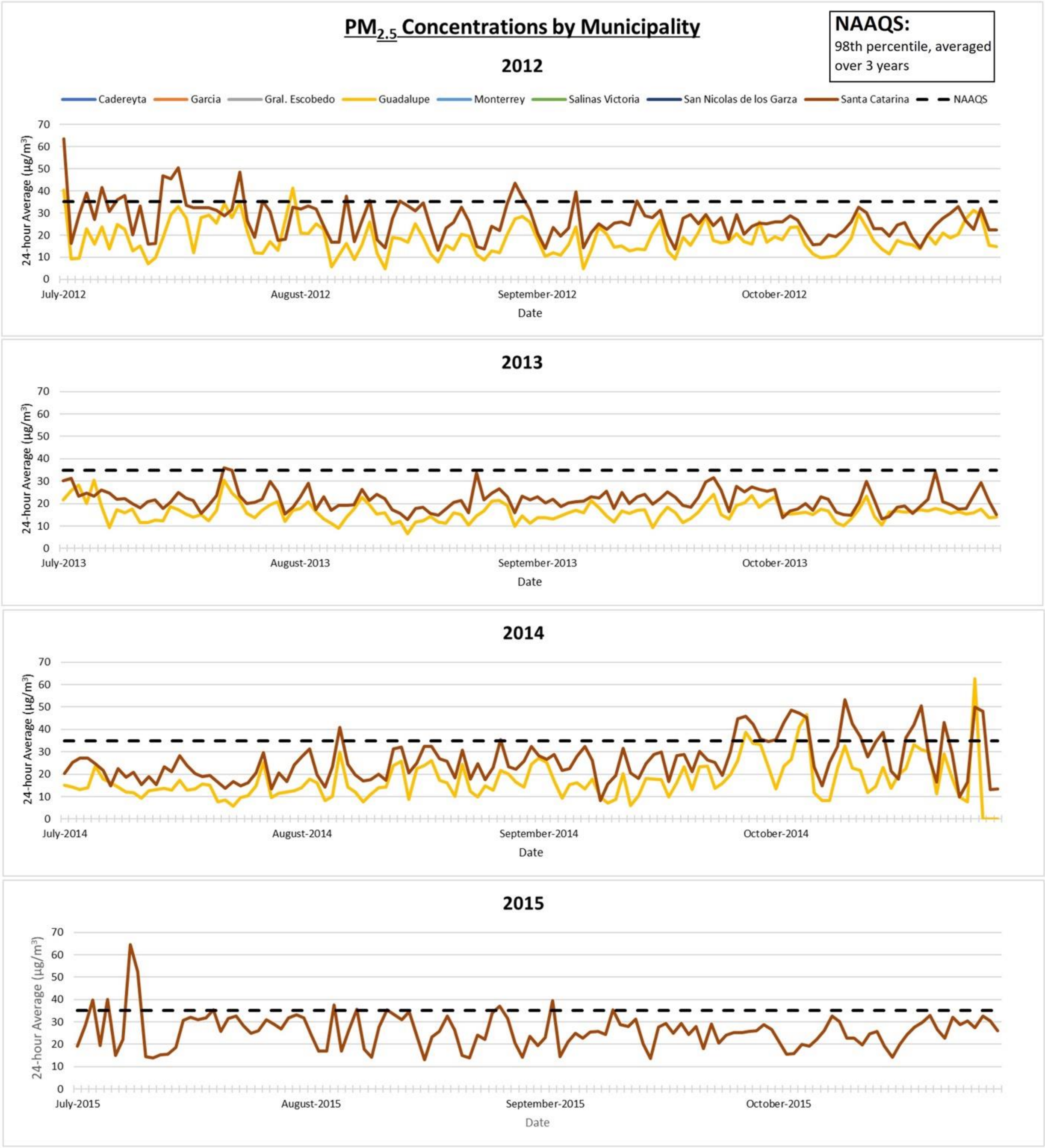

Figure S6. Daily particulate matter ($PM_{2.5}$) concentrations by muncipality. Averaging times are based on the U.S. National Ambient Air Quality Standards [2].

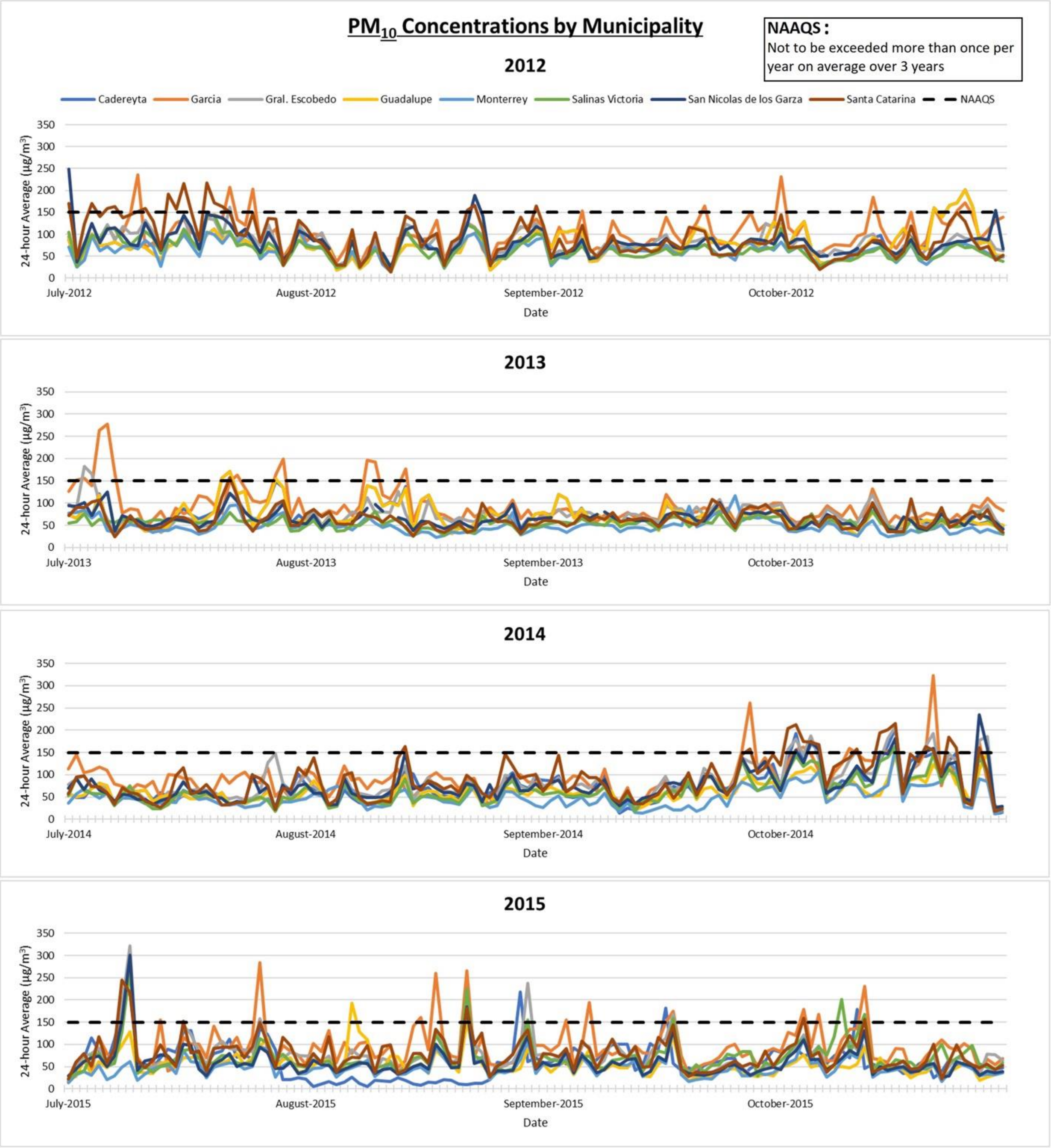

Figure S7. Daily particulate matter (PM_{10}) concentrations by muncipality. Averaging times are based on the U.S. National Ambient Air Quality Standards [2].

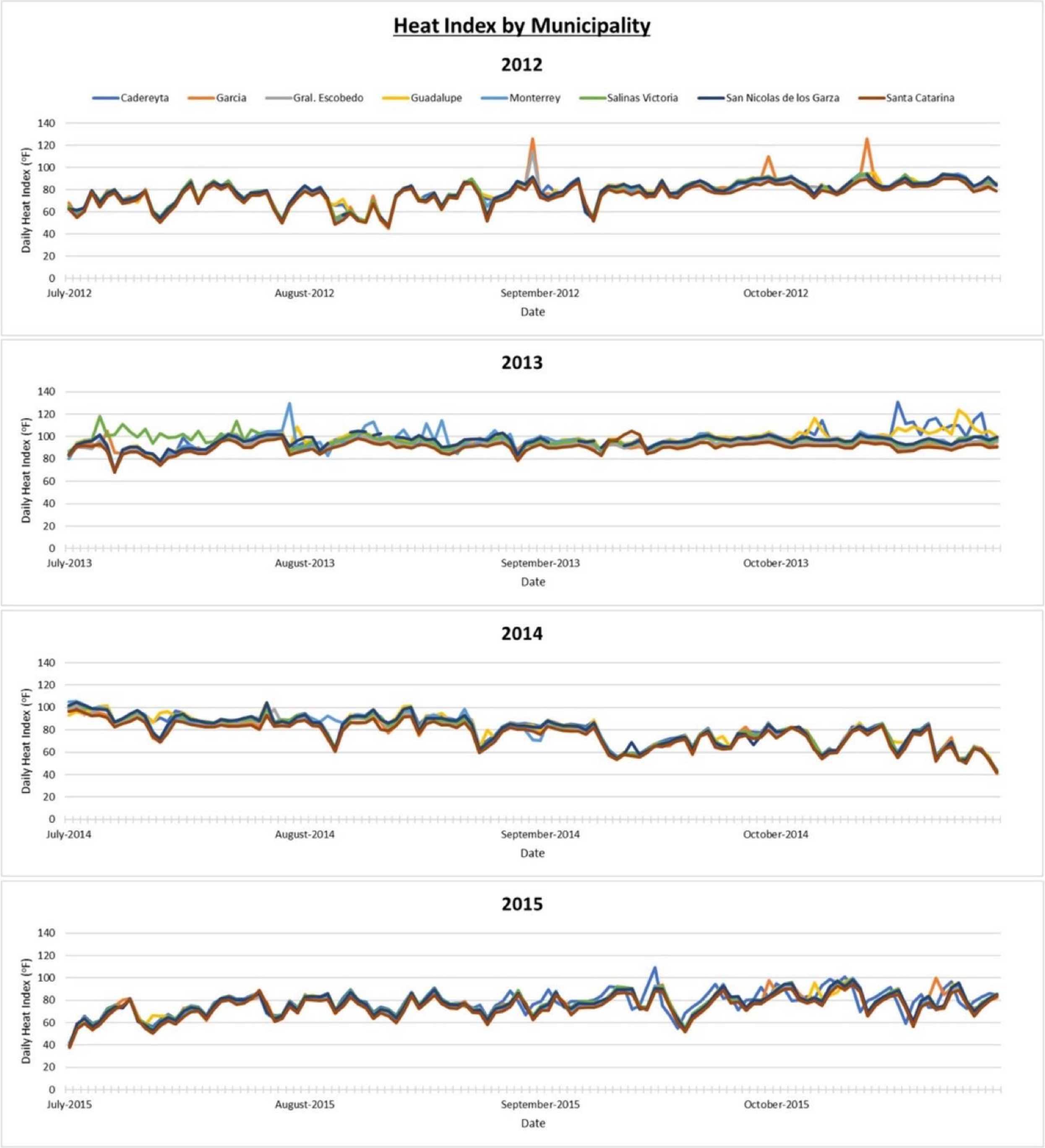

Figure S8. Daily Heat Index (HI) by muncipality.
